# Spatial distribution patterns of human resources allocation in maternal and child healthcare institutions in China from 2016 to 2021

**DOI:** 10.1186/s12913-024-11153-2

**Published:** 2024-06-13

**Authors:** Xiaohui Li, Mei Su, Li He, Jianjun Yang, Fangyuan Wu

**Affiliations:** 1 Guyuan Maternal and Child Healthcare Hospital, Guyuan, Ningxia 756000 China; 2 School of Humanities and Management, Ningxia Medical University, Yinchuan, Ningxia 750001 China; 3 School of Public Health, Ningxia Medical University, Yinchuan, 750001 Ningxia China

**Keywords:** Maternal and child healthcare, Healthcare human resources, Spatial autocorrelation, Spatial distribution, China

## Abstract

**Background:**

In China, economic, urbanization, and policy differences between the eastern and western regions lead to uneven healthcare resources. This disparity is more pronounced in the west due to fewer healthcare personnel per thousand individuals and imbalanced doctor-to-nurse ratios, which exacerbates healthcare challenges. This study examines the spatial distribution of human resources in maternal and child healthcare from 2016 to 2021, highlighting regional disparities and offering insights for future policy development.

**Methods:**

The data were sourced from the “China Health and Family Planning Statistical Yearbook” (2017) and the “China Health and Health Statistics Yearbook” (2018–2022). This study utilized GeoDa 1.8.6 software to conduct both global and local spatial autocorrelation analyses, using China’s administrative map as the base dataset.

**Results:**

From 2016 to 2021, there was an upward trend in the number of health personnel and various types of health technical personnel in Chinese maternal and child healthcare institutions. The spatial distribution of these personnel from 2016 to 2021 revealed clusters characterized as high-high, low-low, high-low and low-high. Specifically, high-high clusters were identified in Guangxi, Hunan, Jiangxi, and Guangdong provinces; low-low in Xinjiang Uygur Autonomous Region and Inner Mongolia Autonomous Region; high-low in Sichuan province; and low-high in Fujian and Anhui provinces.

**Conclusions:**

From 2016 to 2021, there was evident spatial clustering of health personnel and various health technical personnel in Chinese maternal and child healthcare institutions, indicating regional imbalances.

## Background

Maternal and child healthcare institutions are crucial in providing maternal and child health services, promoting child development, and ensuring the safety of pregnant and postpartum women [1–3]. China has a substantial population of women and children, whose health needs are becoming increasingly prominent. The continuous adaptation of fertility policies has further heightened the demand for health personnel within maternal and child healthcare institutions, leading to greater expectations for their technical expertise and distribution [4, 5]. With the evolution of population policies, disparities have emerged in the demographics of maternal and child populations across different regions of China, particularly between the eastern and central-western regions. This highlights the necessity to consider not only the overall quantity but also the regional variations in the allocation of health personnel within maternal and child health institutions [6, 7]. Previous research indicates an uneven distribution of medical and nursing staff within these institutions [8, 9]. Best practices indicate that an optimal doctor-to-nurse ratio of 1:1.25 is effective in meeting diverse service demands and ensuring comprehensive care [2]. With the continuous adjustments in fertility policies, optimizing the distribution of human resources for maternal and child healthcare, particularly focusing on equity across regions, is crucial. This effort is vital for enhancing the capabilities of maternal and child health services [10, 11].

Literature review indicates a relative abundance of studies on healthcare human resources, yet several shortcomings remain. Firstly, existing research tends to prioritize other areas of the health system, with less attention given to maternal and child health. Secondly, previous studies predominantly concentrate on traditional equity analyses, neglecting spatial analyses. Thirdly, there is a greater concentration of research on traditional geographic statistical methods, with relatively fewer studies employing visualization analysis methods. Based on these observations, our study covers the period from 2016 to 2021, and uses provincial-level administrative divisions as the unit of analysis. It utilizes both global and local spatial autocorrelation analyses to examine the spatial distribution characteristics and corresponding spatial correlations of human resources in Chinese maternal and child health institutions. Through visualization analysis methods, we aim to reveal geographical distribution patterns and regional disparities in maternal and child human resources, providing scientific evidence for governmental and related organizations in decision-making processes. This research employs spatial autocorrelation analysis to measure the similarity or correlation among adjacent regions, heiping identify data clustering and dispersion, as well as revealing the spatial patterns and dependencies of geographic phenomena. Additionally, unlike geographic statistical analysis, which only presents numeric results, visualization analysis uses graphical representations to enhance understanding of data structure, patterns, and relationships [12, 13].

## Methods

### Data source

The data used in this study were sourced from two key references: the “China Health and Family Planning Statistical Yearbook” for the year 2017 and the “China Health and Health Statistics Yearbook” spanning from 2018 to 2022. These sources provided comprehensive information regarding the number of health personnel and health technical personnel for maternal and child healthcare human resources across all 31 provinces, municipalities, and autonomous regions of China.

### Spatial autocorrelation analysis

Spatial autocorrelation analysis is a statistical technique employed to analyze spatial data, enabling the computation of spatial correlations related to specific attributes of the analyzed phenomena. This approach is crucial for visualizing and understanding the geographical dispersion of these attributes across different spatial units. The method calculates the spatial correlation between the distribution of data points and their neighboring entities [14]. It encompasses both global and local spatial autocorrelation analyses.

### Global spatial autocorrelation

Global spatial autocorrelation pertains to the spatial characteristics of observed values across the entire area under consideration. The most commonly used indicator for assessing global spatial autocorrelation is Moran’s I Index, which quantifies the interrelationships between elements and yields values within the range of -1 to 1 [15]. A positive Moran’s I value suggests that similar values are clustered together, indicating a positive spatial autocorrelation. Conversely, a negative value implies that dissimilar values are adjacent, indicating negative spatial autocorrelation. A zero value suggests a random spatial pattern, or no autocorrelation, reflecting balanced development. The formula for calculating Moran’s I is shown in Eq. (1):

1 $$ {\rm{Mora}}{{\rm{n}}^\prime }{\rm{s}}\,{\rm{I}} = \frac{n}{{\sum\nolimits_{i = 1}^n {\sum\nolimits_{j = 1}^n {{W_{ij}}} } }} \times \frac{{\sum\nolimits_{i = 1}^n {\sum\nolimits_{j = 1}^n {{W_{ij}}} } ({X_i} - X)({X_j} - X)}}{{\sum\nolimits_{i = 1}^n {{{(Xi - X)}^2}} }}$$

In Eq. (1), X_i_ and X_j_ represent the attribute values at locations i and j respectively, X represents the average value of n location attribute values, and W_ij_ represents the spatial weighting matrix. The Z-test is commonly used to test the existence of spatial autocorrelation among the regions. The calculation of the Z-value is shown in Eq. (2):

2 $$ \text{Z}=\frac{\text{I}-\text{E}\left(\text{I}\right)}{\sqrt{\text{V}\text{A}\text{R}\left(\text{I}\right)}}$$

### Local spatial autocorrelation

When examining the local spatial characteristics of specific attribute values and identifying local units that significantly contribute to the global spatial autocorrelation, local spatial autocorrelation analysis methods are pivotal. These methods include the Local Moran’s I Index, Moran scatterplot, among others [16]. The Moran scatterplot, in particular, is nstrumental for visualizing data in two dimensions, where *W*_*Z*_ represents the spatially weighted average, and *Z* denotes the deviations between observed values and the mean. It should be noted that the calculation of the vector-based Global Moran’s I Index, which is distinct from local indices, is detailed in Eq. (3) [17]:

3 $$ \text{I}=\frac{n}{{S}_{0}}\frac{Z{\prime }{W}_{z}}{Z{\prime }Z} $$

The “spatial lag” variable for each spatial unit is calculated as the spatially weighted average of observed values from neighboring units, according to the spatial weighting matrix. This variable reflects the influence of neighboring units on the attribute values of a specific location and is essential in understanding spatial relationships.

The concept of the Moran scatterplot can be compared to the division into quadrants in a standard coordinate system. In this framework, the first and third quadrants signify positive spatial autocorrelation, reflecting that adjacent locations possess similar attribute values. Similarly, the second and fourth quadrants represent negative spatial autocorrelation, where neighboring locations exhibit dissimilar attribute values. The Moran scatterplot serves as an effective visualization tool, enabling the identification of regions with either similar or dissimilar attributes relative to their neighbors.

The Moran scatterplot concept can be compared to the division into quadrants in a standard coordinate system. In this framework, the first and third quadrants indicate positive spatial autocorrelation, signifying that nearby locations have similar attribute values. Similarly, the second and fourth quadrants denote negative spatial correlation, indicating that nearby locations have dissimilar attribute values. The Moran scatterplot serves as a valuable visualization tool for assessing the spatial autocorrelation patterns within the data, helping to identify regions with similar or dissimilar attributes in relation to their neighbors.

### Data processing and statistical analysis

This study utilized Excel 2016, SPSS 25.0, and GeoDa 1.8.6 software for data processing and statistical analysis. These tools facilitated data organization, descriptive analysis, exploration of temporal trends, and spatial analysis techniques. We collected data from maternal and child healthcare institutions across various regions in China to assess the equity and rationality of the allocation of healthcare human resources.

## Results

### Allocation of Chinese maternal and child healthcare human resources from 2016 to 2021

From 2016 to 2021, a consistent upward trajectory was observed in the number of health personnel and health technical personnel per thousand individuals in Chinese maternal and child healthcare institutions. Specifically, the ratio of health personnel per thousand individuals increased from 0.281 in 2016 to 0.384 in 2021, while the ratio of health technical personnel per thousand individuals rose from 0.189 in 2016 to 0.302 in 2021. Furthermore, the ratio of various health technical personnel per thousand individuals exhibited an increasing pattern, with registered nurses experiencing the most substantial growth. Table 1 presents the specific data.

**Table 1:** The allocation of Chinese maternal and child healthcare human resources from 2016 to 2021

Years	Total population (million)	Number of maternal and child health personnel (*n*, *N*/10^3^population)												
		Health personnel		Health technicians		Practicing physicians		Registered nurses		Pharmacists		Technicians		Doctor-to-nurse ratio
2016	1382.71	388,238	0.281	260,701	0.189	103,360	0.075	138,266	0.099	13,468	0.009	23,154	0.017	1:1.34
2017	1390.08	426,881	0.307	308,712	0.222	113,259	0.081	155,190	0.112	14,508	0.010	25,755	0.019	1:1.37
2018	1405.41	454,985	0.324	346,173	0.246	135,330	0.096	167,702	0.119	15,413	0.011	27,728	0.020	1:1.24
2019	1410.08	486,856	0.345	373,582	0.265	142,879	0.101	184,710	0.131	16,287	0.012	29,706	0.021	1:1.29
2020	1412.12	514,734	0.364	396,480	0.281	152,076	0.107	196,000	0.139	17,204	0.012	31,200	0.022	1:1.29
2021	1412.60	542,332	0.384	426,628	0.302	159,332	0.113	210,259	0.149	18,521	0.013	38,516	0.027	1:1.32
Growth volume	29.89	154,094	0.103	165,927	0.113	55,972	0.038	71,993	0.050	5053	0.004	15,362	0.010	-

Note: ‘Growth volume’ refers to the increase from 2016 to 2021

### Global spatial autocorrelation analysis of health personnel allocation

**Fig. 1:**
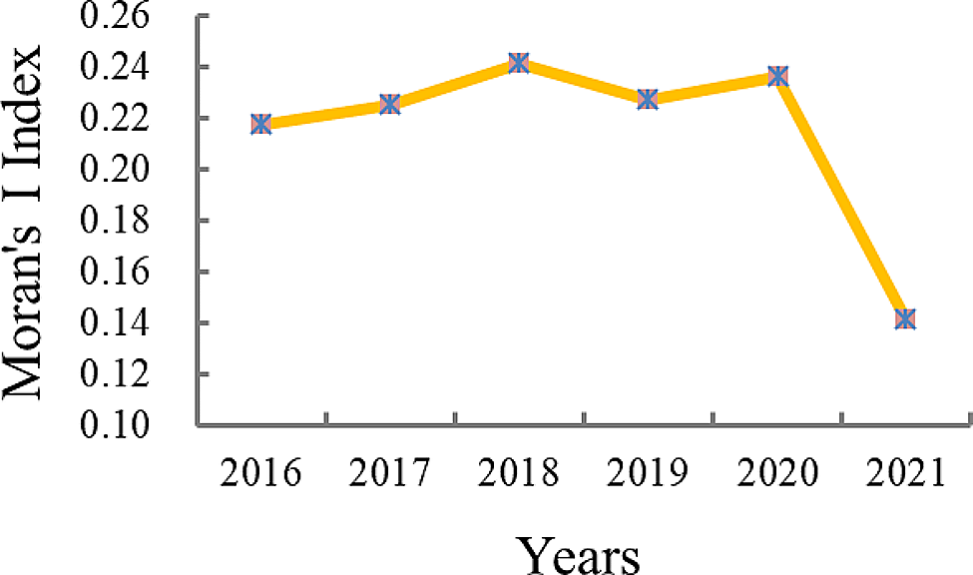
Global Moran’s I index of health personnel in Chinese maternal and child healthcare institutions from 2016 to 2021

From 2016 to 2021, global spatial autocorrelation analysis assessed the distribution of human resource for health within Chinese maternal and child healthcare institutions. The results indicated a range of Moran’s I Index values (0.141 to 0.241) for the allocation of health personnel, all surpassing 0. Statistical significance was confirmed via randomization tests (Z > 1.85, *P* < 0.05), demonstrating a distinct spatial clustering pattern among health personnel distribution. Notably, the highest index value occurred in 2018 (0.241), while the lowest was in 2021 (0.141). Table 2 shows the detailed data. The Moran’s I Index consistently remained high and stable from 2016 to 2020, but notably declined in 2021. Figure 1 provides further insights into this trend.

**Table 2:** Global Moran’s I Index analysis of Chinese maternal and child health personnel from 2016 to 2021

Years	Moran’s I Index	E(I)	Mean	SD	*Z*	*P*
2016	0.217	-0.034	-0.035	0.116	2.163	0.025
2017	0.225	-0.034	-0.037	0.112	2.348	0.015
2018	0.241	-0.034	-0.035	0.113	2.435	0.020
2019	0.228	-0.034	-0.038	0.112	2.354	0.016
2020	0.236	-0.034	-0.038	0.120	2.284	0.019
2021	0.141	-0.034	-0.035	0.095	1.851	0.047

### Global spatial autocorrelation analysis of health technical personnel allocation

**Fig. 2:**
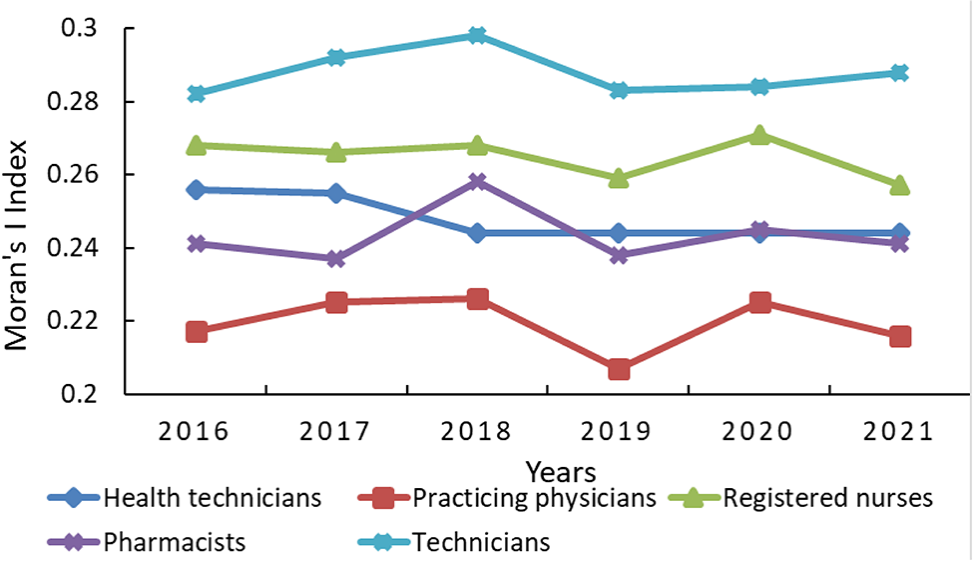
Global Moran’s I Index of the various health technicians in Chinese maternal and child healthcare institutions from 2016 to 2021

We conducted a global spatial autocorrelation analysis to investigate the distribution of diverse health technical personnel in Chinese maternal and child healthcare institutions from 2016 to 2021. The analyses revealed Moran’s I Index values ranging from 0.207 to 0.298, consistently demonstrating positive autocorrelation during this period. Random permutation tests confirmed statistically significant spatial autocorrelation (Z > 2.08, *P* < 0.05), emphasizing a notable spatial clustering pattern in the arrangement of different health technical personnel categories within these institutions. Across the categories, health technical personnel displayed the highest Moran’s I Index in 2016 (0.256). Practicing physicians peaked in 2018 (0.226) and hit their lowest in 2019 (0.207). For registered nurses, the highest Moran’s I Index was noted in 2020 (0.271), with the lowest in 2021 (0.257). Pharmacists reached their peak Moran’s Index in 2018 (0.258), and registered the lowest in 2017(0.237). Technicians reached their highest Moran’s Index in 2018 (0.298), with the lowest observed in 2016 (0.282) Table 3 provides a detailed overview. The spatial clustering of health technical personnel initially declined but subsequently stabilized, indicating a period of consistent growth. Various health technical personnel categories displayed consistent development, with a temporary surge in 2018. Notably, practicing physicians had a lower Moran’s Index compared to other categories. Figure 2 provides a comprehensive overview of these trends.

**Table 3:** Global Moran’s I Index analysis of various types of health technical personnel from 2016 to 2021

Years	Moran’s I Index	E(I)	Mean	SD	*Z*	*P*
**Health technicians**						
2016	0.256	-0.034	-0.038	0.115	2.571	0.011
2017	0.255	-0.034	-0.030	0.117	2.445	0.015
2018	0.244	-0.034	-0.037	0.108	2.605	0.004
2019	0.244	-0.034	-0.033	0.115	2.407	0.018
2020	0.244	-0.034	-0.033	0.114	2.434	0.016
2021	0.244	-0.034	-0.043	0.110	2.627	0.013
**Practicing physicians**						
2016	0.217	-0.034	-0.035	0.117	2.164	0.025
2017	0.225	-0.034	-0.037	0.112	2.348	0.015
2018	0.226	-0.034	-0.030	0.117	2.185	0.025
2019	0.207	-0.034	-0.033	0.116	2.077	0.029
2020	0.226	-0.034	-0.041	0.115	2.314	0.019
2021	0.216	-0.034	-0.033	0.117	2.122	0.031
**Registered nurses**						
2016	0.268	-0.034	-0.032	0.114	2.636	0.012
2017	0.266	-0.034	-0.034	0.115	2.600	0.007
2018	0.268	-0.034	-0.037	0.115	2.661	0.010
2019	0.260	-0.034	-0.036	0.113	2.635	0.011
2020	0.271	-0.034	-0.036	0.113	2.723	0.008
2021	0.257	-0.034	-0.042	0.112	2.662	0.009
**Pharmacists**						
2016	0.241	-0.034	-0.034	0.113	2.446	0.015
2017	0.237	-0.034	-0.034	0.109	2.474	0.016
2018	0.258	-0.034	-0.027	0.112	2.540	0.008
2019	0.239	-0.034	-0.029	0.112	2.386	0.012
2020	0.245	-0.034	-0.038	0.111	2.562	0.010
2021	0.240	-0.034	-0.039	0.112	2.492	0.014
**Technicians**						
2016	0.282	-0.034	-0.035	0.118	2.707	0.012
2017	0.292	-0.034	-0.039	0.114	2.889	0.004
2018	0.298	-0.034	-0.033	0.119	2.780	0.010
2019	0.283	-0.034	-0.279	0.116	2.681	0.011
2020	0.284	-0.034	-0.028	0.119	2.630	0.011
2021	0.288	-0.034	-0.036	0.118	2.742	0.011

### Local spatial autocorrelation analysis of health personnel distribution

**Fig. 3:**
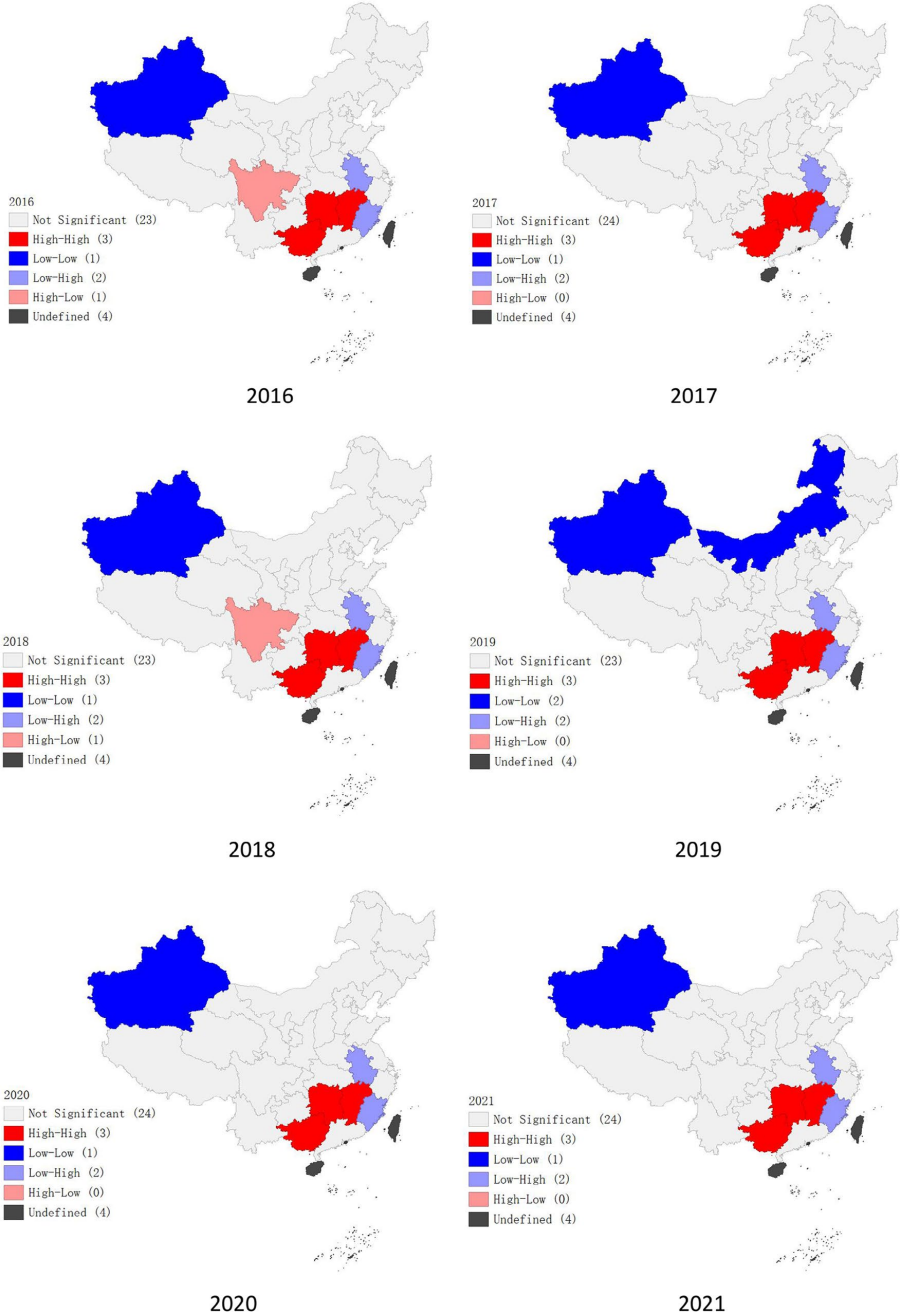
Regional significance in health personnel allocation in Chinese maternal and child healthcare institutions from 2016 to 2021

The local spatial autocorrelation analysis of health personnel in Chinese maternal and child healthcare institutions from 2016 to 2021 revealed distinct spatial patterns. In Guangxi province, Hunan province, and Jiangxi province, there were a significant concentration of health personnel, indicating a high-high pattern of spatial autocorrelation. Conversely, Xinjiang Uygur Autonomous Region exhibited a low-low spatial autocorrelation pattern. Sichuan province showed a combination of high-low spatial autocorrelation in 2016 and 2018, while Fujian province and Anhui province primarily demonstrated a low-high pattern. For further details, please refer to Fig. 3.

### Local spatial autocorrelation analysis of the configuration of various health technical personnel in Chinese maternal and child healthcare institutions from 2016 to 2021

From 2016 to 2021, the health technical personnel in maternal and child healthcare institutions in China exhibited distinct spatial clustering patterns. These patterns were primarily characterized by high-high, low-low, and low-high spatial autocorrelation, with fewer occurrences of high-low clustering. Specifically, high-high clustering of health technical personnel was prominent in Guangxi, Hunan, and Jiangxi provinces. Conversely, Xinjiang Uygur Autonomous Region and Inner Mongolia Autonomous Region displayed significant low-low clustering patterns. Sichuan province was noted for high-low clustering during 2016–2017. Low-high clustering patterns were observed in Fujian and Anhui provinces. Focusing on practicing physicians, significant high-high clustering was similarly observed in Guangxi, Hunan, and Jiangxi, while Xinjiang was characterized by low-low clustering. Sichuan exhibited high-low clustering, and Fujian as well as Anhui showed low-high clustering patterns. For registered nurses, high-high clustering predominated in Guangxi, Hunan, Jiangxi, and Guangdong provinces. Low-low clustering was concentrated in Xinjiang, Inner Mongolia, and Jilin. Fujian and Anhui demonstrated a low-high clustering pattern. Regarding pharmacists and technicians, high-high clustering was noted in Guangxi, Hunan, Jiangxi, and Guangdong. Xinjiang and Inner Mongolia were marked by low-low patterns. Sichuan showed high-low clustering, while Fujian and Anhui exhibited low-high clustering. For a detailed breakdown of these patterns, please refer to Table 4.

**Table 4:** Significance testing of maternal and child health technical personnel allocation in China from 2016 to 2021

Years	High-high	High-low	Low-high	Low-low
**Health technicians**				
2016	Guangxi ^*^、Hunan ^*^、Jiangxi ^*^、Guangdong^*^	Sichuan ^*^	Fujian ^*^、Anhui ^*^	Xinjiang ^**^
2017	Guangxi ^*^、Hunan ^*^、Jiangxi ^*^、Guangdong^*^	Sichuan ^*^	Fujian ^*^、Anhui ^*^	Xinjiang ^**^、Inner Mongolia^*^
2018	Guangxi ^*^、Hunan ^*^、Jiangxi ^*^		Fujian ^*^、Anhui ^*^	Xinjiang ^*^、Inner Mongolia^*^
2019	Guangxi ^*^、Hunan ^*^、Jiangxi ^*^		Fujian ^*^、Anhui ^*^	Xinjiang ^*^、Inner Mongolia^*^
2020	Guangxi ^*^、Hunan ^*^、Jiangxi ^*^		Fujian ^*^、Anhui ^*^	Xinjiang ^*^、Inner Mongolia^*^
2021	Guangxi ^*^、Hunan ^*^、Jiangxi ^*^		Fujian ^*^、Anhui ^*^	Xinjiang ^*^、Inner Mongolia^*^
**Practicing physicians**				
2016	Guangxi ^*^、Hunan ^*^、Jiangxi ^*^	Sichuan ^*^	Fujian ^*^、Anhui ^**^	Xinjiang ^**^
2017	Guangxi ^*^、Hunan ^*^、Jiangxi ^*^	Sichuan ^*^	Fujian ^*^、Anhui ^**^	Xinjiang ^**^
2018	Guangxi ^*^、Hunan ^*^、Jiangxi ^*^	Sichuan ^*^	Fujian ^*^、Anhui ^**^	Xinjiang ^**^
2019	Guangxi ^*^、Jiangxi ^*^	Sichuan ^*^	Fujian ^*^、Anhui ^**^	Xinjiang ^**^
2020	Guangxi ^*^、Hunan ^*^、Jiangxi ^*^	Sichuan ^*^	Fujian ^*^、Anhui ^**^	Xinjiang ^**^
2021	Guangxi ^*^、Hunan ^*^、Jiangxi ^*^、Shandong^*^	Sichuan ^*^	Fujian ^*^、Anhui ^**^	Xinjiang ^*^、Inner Mongolia^*^
**Registered nurses**				
2016	Guangxi ^*^、Hunan ^*^、Jiangxi ^*^、Guangdong^*^	Sichuan ^*^	Fujian ^*^、Anhui ^*^	Xinjiang ^**^、Inner Mongolia^*^
2017	Guangxi ^*^、Hunan ^*^、Jiangxi ^*^、Guangdong^*^	Sichuan ^*^	Fujian ^*^、Anhui ^*^	Xinjiang ^*^、Inner Mongolia^*^
2018	Guangxi ^*^、Hunan ^*^、Jiangxi ^*^、Guangdong^*^		Fujian ^*^、Anhui ^*^	Xinjiang ^**^、Inner Mongolia^*^
2019	Guangxi ^*^、Hunan ^*^、Jiangxi ^*^、Guangdong^*^		Fujian ^*^、Anhui ^*^	Xinjiang ^*^、Inner Mongolia^*^、Jilin^*^
2020	Guangxi ^*^、Hunan ^*^、Jiangxi ^*^、Guangdong^*^		Fujian ^*^、Anhui ^*^	Xinjiang ^*^、Inner Mongolia^*^、Jilin^*^
2021	Guangxi ^*^、Hunan ^*^、Jiangxi ^*^		Fujian ^*^、Anhui ^**^	Xinjiang ^*^、Inner Mongolia^*^、Jilin^*^
**Pharmacists**				
2016	Guangxi ^*^、Hunan ^*^、Jiangxi ^*^、Guangdong^*^	Sichuan ^*^	Fujian ^*^、Anhui ^*^	Xinjiang ^**^
2017	Guangxi ^*^、Hunan ^*^、Jiangxi ^*^、Guangdong^*^	Sichuan ^*^	Fujian ^*^、Anhui ^*^	Xinjiang ^**^
2018	Guangxi ^*^、Hunan ^*^、Jiangxi ^*^、Guangdong^*^	Sichuan ^*^	Fujian ^**^、Anhui ^*^	Xinjiang ^**^、Inner Mongolia^*^
2019	Guangxi ^*^、Hunan ^*^、Jiangxi ^*^、Guangdong^*^	Sichuan ^*^	Fujian ^**^、Anhui ^*^	Xinjiang ^**^、Inner Mongolia^*^
2020	Guangxi ^*^、Hunan ^*^、Jiangxi ^*^、Guangdong^*^		Fujian ^**^、Anhui ^*^	Xinjiang ^*^、Inner Mongolia^*^
2021	Guangxi ^*^、Hunan ^*^、Jiangxi ^*^、Guangdong^*^		Fujian ^**^、Anhui ^*^	Xinjiang ^*^、Inner Mongolia^*^
**Technicians**				
2016	Guangxi ^*^、Hunan ^*^、Jiangxi ^*^、Guangdong^*^	Sichuan ^*^	Fujian ^*^、Anhui ^*^	Xinjiang ^**^
2017	Guangxi ^*^、Hunan ^*^、Jiangxi ^*^、Guangdong^*^	Sichuan ^*^	Fujian ^*^、Anhui ^*^	Xinjiang ^**^
2018	Guangxi ^*^、Hunan ^*^、Jiangxi ^*^、Guangdong^*^	Sichuan ^*^	Fujian ^*^、Anhui ^**^	Xinjiang ^**^、Inner Mongolia^*^
2019	Guangxi ^*^、Hunan ^*^、Jiangxi ^*^、Guangdong^*^		Fujian ^*^、Anhui ^**^	Xinjiang ^*^、Inner Mongolia^*^
2020	Guangxi ^*^、Hunan ^*^、Jiangxi ^*^、Guangdong^*^		Fujian ^*^、Anhui ^*^	Xinjiang ^*^、Inner Mongolia^*^
2021	Guangxi ^*^、Hunan ^*^、Jiangxi ^*^、Guangdong^*^		Fujian ^*^、Anhui ^**^	Xinjiang ^*^、Inner Mongolia^*^、Jilin^*^

Note: ^*^ indicates areas with *P* < 0.05 and ^**^ denotes areas with *P* < 0.001

## Discussion

### Chinese maternal and child healthcare human resources show an increasing trend

The steady increase in the number of human resources in Chinese maternal and child healthcare institutions represents a positive trend. From 2016 to 2021, this study observed an uptrend in both health personnel and various categories of health technicians per thousand population. Despite these yearly increments, a persistent inadequacy in the overall quantity of health personnel remains. This conclusion was supported by estimates from Chinese scholars in 2021, which indicated a demand for approximately 520,000 health technicians in these institutions, while the current deployment was only about 426,030, highlighting a consistently supply-demand imbalance [18]. Our study also noted fluctuations in the doctor-to-nurse ratio of Chinese maternal and child healthcare human resources from 2016 to 2021, ranging from 1:1.24 to 1:1.37. Nevertheless, the ratio reached the target set by the “National Health Service System Plan (2015–2020)” for the year 2020, which was 1:1.25, for most of the five-year period [19]. This suggests that despite some fluctuations, the doctor-to-nurse ratio remained generally reasonable. However, in the context of changing population policies and uneven economic development, variations in the demand for human resources arise across different regions. It is imperative to further focus on the strategic allocation of human resources in different regional maternal and child healthcare institutions.

### Spatial distribution patterns and their implications

This study illustrated discernible spatial agglomeration characteristics in the distribution of Chinese maternal and child healthcare human resources. Notably, the Moran’s I Index for health personnel was consistently high and stable from 2016 to 2020, but experienced a significant decline in 2021 (*P* < 0.05). This trend suggests an improvement in spatial concentration by 2021, which may reflect positive developments in resource allocation and distribution. This positive trend could potentially be attributed to the implementation of two significant policies in Chinese maternal and child healthcare, namely the “Maternal and Child Safety Action Plan (2018–2020)” and the “Healthy Children Action Plan (2018–2020).” Furthermore, the Moran’s I Index for health technicians consistently remained at 0.244 from 2018 to 2021. This observation indicates that the distribution pattern of health technicians among different regions has remained relatively stable in recent years, with a concentration in specific geographical areas. This may be attributed to the fact that healthcare technicians are a core component of the human resources in maternal and child healthcare institutions. The stability of these technicians is crucial for ensuring the continuity and quality of medical services, which likely results in them being given priority in terms of job protection. Notably, among the diverse health technical personnel, the Moran’s I Index for practicing physicians consistently exhibited lower values compared to other health technical personnel, indicating less spatial clustering and smaller regional disparities. This finding suggests relatively good geographic equity among practicing physicians, aligning with the research findings of Ma et al. [2]. In contrast, the Moran’s Index for technicians ranged from 0.282 to 0.288, surpassing the values observed for other categories of technical personnel across different years. This suggests noticeable spatial concentrations and regional disparities in the distribution of technical personnel, which may influence the overall geographic equity of maternal and child healthcare human resources. The observed differences in spatial clustering between these two categories of health technical personnel can be attributed to their distinct roles within health institutions. Furthermore, the distribution of technicians is influenced by various factors, including regional economic development, health requirements, and institutional size. In developed regions, maternal and child healthcare institutions tend to employ a greater number of technicians, whereas underdeveloped regions face a scarcity [20–22]. Therefore, to improve the equity of maternal and child health services, it is imperative to optimize the geographical distribution of practicing physicians, registered nurses, and technicians. Balancing their allocation will be vital to ensuring that maternal and child health services are accessible and equitable across all regions.

### Regional clustering of Chinese maternal and child healthcare human resources allocation

In China, the allocation of maternal and child healthcare human resources displays a discernible pattern of clustering in both hotspots and coldspots, resulting in significant regional differences. Previous research has identified the issue of inequitable and disproportionate distribution of health personnel in Chinese maternal and child healthcare institutions [2]. Our study further shed light on the notable spatial clustering pattern of health personnel and diverse health technical staff within Chinese maternal and child healthcare institutions across different provinces during the period from 2016 to 2021. Specifically, Guangxi, Hunan, Jiangxi, and Guangdong provinces presented a pronounced high-high clustering phenomenon in the allocation of maternal and child healthcare human resources. These provinces may have a higher demand for human resources in maternal and child healthcare institutions due to their higher population density, and they may also prioritize the allocation of these resources [23]. Conversely, Xinjiang and Inner Mongolia exhibited a low-level clustering pattern for maternal and child healthcare human resources. This clustering is influenced by several factors, including their relatively underdeveloped economies, lagging progress in maternal and child health services, incomplete institutional frameworks, and insufficient support mechanisms [24]. Sichuan province displayed a clustering pattern characterized by a high-low resource allocation combination. This trend can be attributed to its robust economic growth, higher population density, and ample health resources. Consequently, there is a relatively abundant distribution of health resources in maternal and child healthcare institutions in Sichuan, effectively fulfilling the strategic goal of a strong health workforce [25]. In contrast, provinces like Fujian and Anhui showed a low-high resource allocation clustering pattern. This pattern suggests a lower concentration of resources within these provinces but a higher allocation in neighboring provinces. This may be because neighboring provinces, which are often more economically developed and densely populated, tend to have more abundant healthcare human resources. However, Anhui and Fujian provinces have relatively smaller populations, resulting in lower demand for healthcare human resources. This phenomenon has led to the emergence of spatial clustering characterized by a low-high pattern.

## Conclusions

In general, from 2016 to 2021, there has been a steady increase in the quantity of Chinese maternal and child healthcare human resources. However, significant imbalances in the distribution of these resources have been identified, particularly manifesting as spatial clustering. In regions that are economically developed and densely populated, the availability of maternal and child healthcare professionals is relatively sufficient. Conversely, in the underdeveloped and sparsely populated central and western regions, there is a prevalent shortage of health personnel. To address these disparities, we propose enhancing the training and distribution of Chinese maternal and child healthcare human resources, paying particular attention to regions with insufficient health personnel.

## Data Availability

The data used in this study are sourced from the “China Health and Family Planning Statistics Yearbook” in 2017 (http://www.nhc.gov.cn) and survey data from the “China Health Statistics Yearbook” in 2018-2022 (http://www.stats.gov.cn/).

## References

[1] Zhou M, Zhang L, Hu N (2020). Association of primary care physician supply with maternal and child health in China: a national panel dataset, 2012–2017. BMC Public Health.

[2] Ma Y, Xiao P, Yu L (2023). The allocation and fairness of human resource for health in Chinese maternal and child health care institutions: a nationwide longitudinal study. BMC Health Serv Res.

[3] Ren Z, Song P, Theodoratou E (2015). China’s human resources for maternal and child health: a national sampling survey. BMC Health Serv Res.

[4] Gao Y, Zhou H, Singh NS (2017). Progress and challenges in maternal health in western China: a countdown to 2015 national case study. Lancet Glob Health.

[5] Tatum M (2021). China’s three-child policy. Lancet.

[6] Xu L, Yang F, Sun J (2019). Evaluating family planning organizations under China’s two-child policy in Shandong province. Int J Environ Res Public Health.

[7] Zang S, OuYang J, Zhao M (2021). Factors associated with child delivery expenditure during the transition to the national implementation of the two-child policy in China. Health Qual Life Outcomes.

[8] Erdenee O, Paramita SA, Yamazaki C, Koyama H (2017). Distribution of health care resources in Mongolia using the Gini coefficient. Hum Resour Health.

[9] Guo Y, Huang Y (2019). Realising equity in maternal health: China’s successes and challenges. Lancet.

[10] Matsumoto K, Seto K, Hayata E (2021). The geographical maldistribution of obstetricians and gynecologists in Japan. PLoS ONE.

[11] Chen Y (2013). New approaches for calculating Moran’s index of spatial autocorrelation. PLoS ONE.

[12] Ranganathan P (2021). An introduction to statistics: choosing the correct statistical test. Indian J Crit Care Med.

[13] Yuan X, Yan J, Sun L (2023). The influence o f presentation frames of visualization information for safety on situational awareness under a three-level user-interface design. Int J Environ Res Public Health.

[14] Raza O, Mansournia MA, Rahimi Foroushani A (2020). Exploring spatial dependencies in the prevalence of childhood diarrhea in Mozambique using global and local measures of spatial autocorrelation. Med J Islamic Repub Iran.

[15] Chen Y (2021). An analytical process of spatial autocorrelation functions based on Moran’s index. PLoS ONE.

[16] Lozada A, Bertin A (2022). Spatial autocorrelation signatures of ecological determinants on plant community characteristics in high Andean wetlands. Sci Rep.

[17] Zhang H, Yang L, Li L (2019). The epidemic characteristics and spatial autocorrelation analysis of hand, foot and mouth disease from 2010 to 2015 in Shantou, Guangdong, China. BMC Public Health.

[18] Chen L, Zhao Z, Liu Y (2024). Research on the measurement of human resource for health demand and its spatiotemporal pattern in national maternal and child health care institutions. Mod Prev Med.

[19] Yu H, Yu S, He D (2021). Equity analysis of Chinese physician allocation based on Gini coefficient and theil index. BMC Health Serv Res.

[20] Huang M, Luo D, Wang Z (2020). Equity and efficiency of maternal and child health resources allocation in Hunan Province, China. BMC Health Serv Res.

[21] Bai Q, Ke X, Huang L, Liu L, Xue D, Bian Y (2022). Finding flaws in the spatial distribution of health workforce and its influential factors: an empirical analysis based on Chinese provincial panel data, 2010–2019. Front Public Health.

[22] Renfrew MJ, McFadden A, Bastos MH (2014). Midwifery and quality care: findings from a new evidence-informed framework for maternal and newborn care. Lancet.

[23] Wu J, Yang Y (2019). Inequality trends in the demographic and geographic distribution of health care professionals in China: data from 2002 to 2016. Int J Health Plann Manage.

[24] Wang Z, He H, Liu X (2023). Health resource allocation in Western China from 2014 to 2018. Arch Public Health.

[25] Gong J, Shi L, Wang X, Sun G (2023). The efficiency of health resource allocation and its influencing factors: evidence from the super efficiency slack based model-tobit model. Int Health.

